# Potential Role of the Last Half Repeat in TAL Effectors Revealed by a Molecular Simulation Study

**DOI:** 10.1155/2016/8036450

**Published:** 2016-10-10

**Authors:** Hua Wan, Shan Chang, Jian-ping Hu, Xu-hong Tian, Mei-hua Wang

**Affiliations:** ^1^College of Mathematics and Informatics, South China Agricultural University, Guangzhou, China; ^2^School of Electrical and Information Engineering, Jiangsu University of Technology, Changzhou, China; ^3^Faculty of Biotechnology Industry, Chengdu University, Chengdu, China

## Abstract

TAL effectors (TALEs) contain a modular DNA-binding domain that is composed of tandem repeats. In all naturally occurring TALEs, the end of tandem repeats is invariantly a truncated half repeat. To investigate the potential role of the last half repeat in TALEs, we performed comparative molecular dynamics simulations for the crystal structure of DNA-bound TALE AvrBs3 lacking the last half repeat and its modeled structure having the last half repeat. The structural stability analysis indicates that the modeled system is more stable than the nonmodeled system. Based on the principle component analysis, it is found that the AvrBs3 increases its structural compactness in the presence of the last half repeat. The comparison of DNA groove parameters of the two systems implies that the last half repeat also causes the change of DNA major groove binding efficiency. The following calculation of hydrogen bond reveals that, by stabilizing the phosphate binding with DNA at the C-terminus, the last half repeat helps to adopt a compact conformation at the protein-DNA interface. It further mediates more contacts between TAL repeats and DNA nucleotide bases. Finally, we suggest that the last half repeat is required for the high-efficient recognition of DNA by TALE.

## 1. Introduction

Transcriptional activator-like effectors (TALEs) are DNA-binding proteins secreted by* Xanthomonas* bacteria [1]. In TALEs, the DNA-binding domain is composed of a repeated highly conserved 33~35 (mostly 34) amino acids' sequence with the exception of the 12th and 13th amino acids. These two residues, known as repeat-variable diresidues (RVDs), are responsible for the specific nucleotide recognition [2, 3]. Both experimental [2] and computational [3] studies found that there is a strong correlation between RVDs and target DNA bases. For example, RVDs Asn/Ile (NI), His/Asp (HD), and Asn/Gly (NG) recognize adenine (A), cytosine (C), and thymine (T), respectively. This simple code allows the design of specific TALE protein by selecting a combination of repeats with appropriate RVDs [4, 5]. The modularity of DNA-binding domain of TALEs has been widely used in biotechnological applications [5, 6], such as genome editing in plants, animals, and human cells, as well as to induce gene expression.

To understand the modular nature of TALE-DNA binding, a series of studies focused on the structural basis for TALE-DNA recognition. In 2010, a nuclear magnetic resonance (NMR) structure of TALE protein PthA was solved by Murakami et al. [7]. The NMR analysis revealed that there are two *α* antiparallel helices in each repeat. In 2012, researchers led by Shi and Yan crystallized two structures of 11.5-repeat TALE dHax3 in the presence and the absence of DNA at resolutions of 1.8 Å and 2.4 Å, respectively [8]. This study uncovered that amino acid 13 of RVD specifies the identity of a DNA base while amino acid 12 of RVD stabilizes the repeat structure. Separately, researchers led by Stoddard determined the 3.0 Å structure of the naturally occurring TALE PthXo1 bound to DNA [9]. This structure contains over 20 repeats, showing examples of the six most common RVD types. In 2013, Stella et al. reported the crystal structure of TALE AvrBs3 in complex with its target DNA, with the last half repeat being unresolved [10]. This study shows a new interaction mode of the initial thymine T_0_ recognition by TALE protein. Additionally, several studies investigated the specificities and efficiencies of TALE-DNA binding [11–13]. The above biochemical data is important for exploring the TALE-DNA recognition mechanism.

Furthermore, theoretical studies also improved our understanding of TALE-DNA interactions. Moscou and Bogdanove used a computational method to decide the TALE recognition code [3]. Bradley modeled the structure of TALE in complex with DNA based on the Rosetta package and successfully predicted the TALE-DNA interaction [14]. Grau et al. developed a new software platform for predicting TAL effector target sites based on a statistical model [15]. Several molecular simulation studies were applied to investigate the specificities of TALE-DNA binding and conformational changes of TALE [16–19]. Nevertheless, some interesting issues still need to be further probed. In all natural TALEs, surprisingly, the last repeat of tandem repeats is always a truncated half repeat [1]. The previous crystallographic data [8] and our molecular simulation study [17] showed that the last repeat of TALE protein dHax3 forms a stable interaction with DNA. It suggests a necessity of the last half repeat for biological functions. However, the last half repeat was also considered to be dispensable for the function of gene activation by both transient expression assays in* Nicotiana benthamiana* and gene-specific targeting in the rice genome [20]. In order to reduce the complexity and costs, the last half repeat was suggested to be omitted in the design of TALE nucleases [20]. Then, is there the necessity for the last half repeat to occur in TALEs? If yes, how does the last half repeat affect the TALE-DNA binding in detail? What is the difference of the protein-DNA interaction between the two DNA-bound TALE proteins, lacking and having the last half repeat?

In order to answer the above questions, we selected the crystal structure of TALE AvrBs3 (lacking the last half repeat) to perform the comparative molecular dynamics (MD) simulations. The two simulated systems, in the absence and the presence of the last half repeat, were built. By performing MD simulations, we compared the stabilities of the two systems. Principal component analysis (PCA) was applied to probe the functional dynamics in the two systems. The groove deformation of TALE-bound DNA was analyzed at the base pair level. To explain the conformational difference between the two systems, we investigated the specific and nonspecific interactions at the TALE-DNA interface. Finally, we proposed the potential role of the last half repeat in the specific recognition and binding of TALE-DNA.

## 2. Systems and Methods

### 2.1. The Structures of AvrBs3-DNA Complex Systems

**Figure 1:**
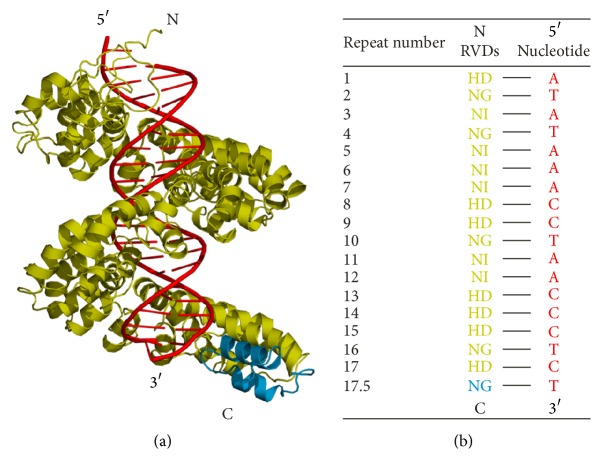
Complex structure and domain organization of AvrBs3 bound to DNA. (a) The complex structure of AvrBs3-DNA. In the crystal structure, AvrBs3 (yellow) contains a 17.5-repeat domain to mediate the DNA (red) binding. The unresolved last half repeat R_17.5_ was modeled based on the last half repeat in the dHax3-DNA structure (PDB codes: 3V6T) and was colored in blue separately. (b) The 17.5-repeat domain of AvrBs3 conferring DNA sequence. In each repeat, RVD residues are responsible for the specific nucleotide recognition of the DNA sense strand.

The crystal structure of the AvrBs3-DNA complex (PDB codes: 2YPF) was obtained from the Protein Data Bank [10]. In the crystal structure, AvrBs3 (yellow) contains a 17.5-repeat TALE domain to confer DNA sequence (red) specificity (Figure 1(a)), with the last half repeat R_17.5_ being unresolved. Then, repeat R_17.5_ (blue) was modeled based on the last half repeat in the TALE dHax3-DNA structure (PDB codes: 3V6T) [8]. A total of 17.5 repeats form a superhelix and bind with the sense strand along the DNA major groove. In each repeat, the RVDs are responsible for recognizing one specific nucleotide (Figure 1(b)). For convenience, the two systems lacking and having repeat R_17.5_ were referred to as the nonmodeled and the modeled systems, respectively.

### 2.2. Molecular Dynamics Simulation

Two independent simulation systems were prepared using VMD 1.9 [21]. In each system, the complex structure was solvated in a periodic box filled with TIP3P water molecules. The minimum distance is about 10 Å from the solute unit to the box wall. Each of the two systems was neutralized by adding 49 sodium ions (Na^+^) with VMD 1.9. Then, the two MD simulations were performed with the NAMD 2.9 program [22] using the CHARMM27 all-atom additive force field for nucleic acids [23]. The SHAKE algorithm [24] was used to constrain all bonds involving hydrogen atoms, and particle mesh Ewald (PME) method [25] was applied to evaluate electrostatic interactions. Meanwhile, Lennard-Jones potential was truncated at a cut-off distance of 12 Å. Each simulation included two stages. (i) The systems were minimized with 20000-step energy minimization and then slowly were heated from 0 to 310 K over 0.5 ns. To keep the stabilization of systems, all backbone atoms of protein and DNA were restrained with a harmonic constant of 0.1 kcal · mol^−1^ · Å^−2^. (ii) After the positional constraints were removed, the productive MD simulations were run for 15 ns under constant pressure (1 atm) and temperature (310 K) conditions. The pressure and temperature were kept using the Langevin piston method [26]. The atomic coordinates were stored every 2.0 ps. Hence, 7500 snapshots in each system were collected for further analysis.

### 2.3. Principal Component Analysis

Principal component analysis (PCA) is a standard method for obtaining a brief picture of motions. This method exacts the highly correlated fluctuations from the MD trajectories through dimensionality reduction. The definition of PCA is based on the construction and diagonalization of the covariance matrix. The element *C*_*ij*_ in the matrix is calculated according to [27]

(1) $$\begin{array}{c} C_{ij}=\langle \;\left(\;x_{i}-\langle \;x_{i}\;\rangle \;\right)\left(\;x_{j}-\langle \;x_{j}\;\rangle \;\right)\;\rangle , \end{array}$$

where *x*_*i*_(*x*_*j*_) is the coordinate of the *i*th (*j*th) atom of the systems and 〈⋯〉 represents an ensemble average. The eigenvectors of the matrix give the directions of the concerted motions. The eigenvalues indicate the magnitude of the motions along the direction. The first few principal components (PCs) usually contain the most important conformational changes of a biomolecular system [17, 28, 29]. In this study, PCA was performed with Gromacs 4.5 package [30] to detect the conformational difference between the two systems.

### 2.4. Conformational Analysis of Nucleic Acids

Curves program is the most widely used in analysis of nucleic acid conformations [31]. This program can provide an entire set of DNA structural parameters. By using the Curves program, we obtain the groove parameters to describe the DNA groove deformation in this paper.

## 3. Results and Discussion

### 3.1. MD Results

**Figure 2:**
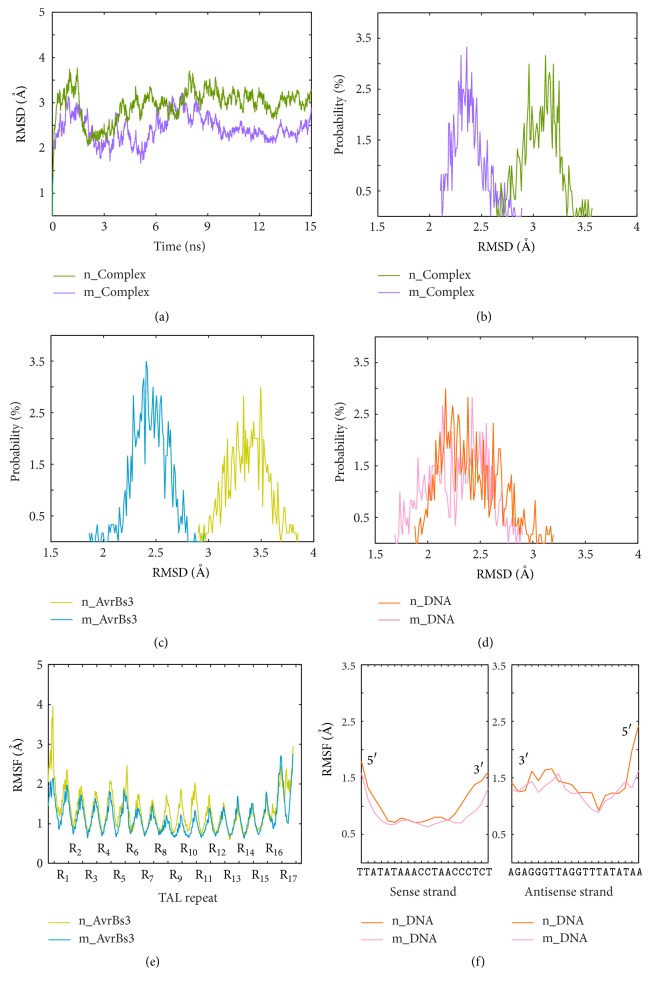
Comparative MD analysis of the nonmodeled system (n_Complex: green; n_AvrBs3: yellow; n_DNA: orange) and the modeled system (m_Complex: purple; m_AvrBs3: blue; m_DNA: pink). (a) The RMSDs of the AvrBs3 backbone atoms versus simulation time. (b~d) The RMSD probability distribution of the AvrBs3-DNA complex (b), AvrBs3 (c), and DNA (d) calculated from the equilibrium trajectories. (e) The RMSFs of the C*α* atoms of AvrBs3 calculated from the equilibrium trajectories. (f) The RMSFs of the P atoms in the sense strand (left) and the antisense strand (right) of DNA calculated from the equilibrium trajectories.

Two 15 ns MD simulations were carried out for the nonmodeled (lacking the last half repeat) and the modeled (having the last half repeat) systems, respectively.  Figure 2(a) compares the root mean square deviation values (RMSDs) of backbone atoms of the AvrBs3-DNA complex from the two systems. The two systems remain relatively stable after 9 ns, and then the last 6 ns MD trajectories are taken as the equilibrium portions for the two systems. Figures 2(b), 2(c), and 2(d) display the distributional probability of RMSD from the equilibrium trajectories. In the nonmodeled system, the RMSDs converge to about 3.07 Å, 3.37 Å, and 2.40 Å for the AvrBs3-DNA complex, AvrBs3, and DNA, respectively. In the modeled system, the RMSDs converge to about 2.38 Å, 2.44 Å, and 2.29 Å for the AvrBs3-DNA complex, AvrBs3, and DNA, respectively. This indicates that the modeled system is more stable than the nonmodeled system. The only difference between the two systems is that the modeled system has an additional repeat, R_17.5_. The previous crystallographic data revealed that the last half repeat contributes to the protein-DNA binding in the structure of DNA-bound TALE dHax3 [17]. All these suggest that the last half repeat increases the structural stability.

We also calculated the root mean square fluctuation values (RMSFs) of the common 17 repeats (from repeat 1 to repeat 17) of AvrBs3 and 20 bases (from position −1 to position 18) of DNA in the two systems from the equilibrium trajectories. The results are given in Figures 2(e) and 2(f), and 17 repeats are labeled as R_1_ to R_17_. In each system, the linker between two adjacent TAL repeats shows higher RMSFs (Figure 2(e)). The RVD loop within each repeat has lower RMSFs because the RVD loop region is the DNA-binding site in a repeat. Of all the repeats, R_17_ undergoes the highest fluctuations. Notably, in the nonmodeled system, the RMSFs of the RVD loop of R_17_ increase markedly relative to the other RVD loops. However, in the modeled system, the RVD loop of R_17_ still maintains relatively lower RMSFs. Meanwhile, the 3′ end of the DNA sense strand is more flexible in the nonmodeled system compared with the modeled system (Figure 2(f)). It indicates that the AvrBs3 of the modeled system is well constrained by DNA. In contrast, the nonmodeled system loses some important protein-DNA contacts. The RMSFs analysis implies that the absence of the last half repeat will partially impair the binding of AvrBs3 to DNA.

### 3.2. Conformational Change of AvrBs3

**Figure 3:**
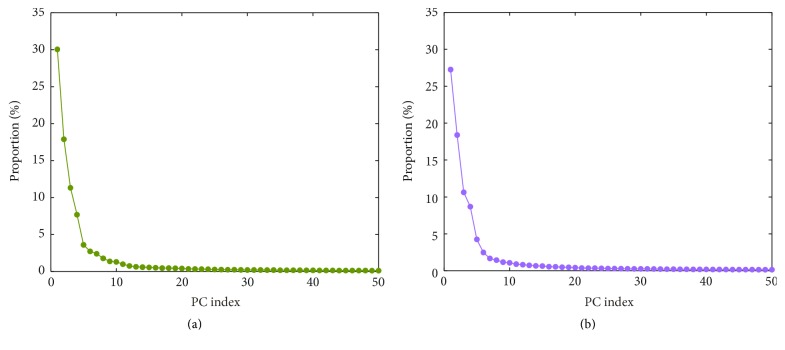
The proportion of system's variance accounted for by the first 50 PCs of the nonmodeled system (a) and the modeled system (b).

Previous studies revealed the conformational plasticity of TALEs bound to DNA [7, 8, 17]. To detect the conformational change of DNA-bound AvrBs3, the PCA was performed for C*α* atoms of protein and P atoms of DNA to obtain slow motions based on the equilibrium trajectories of the nonmodeled and the modeled systems.  Figure 3 gives the proportion of system's variance accounted for by the first 50 PCs, which was calculated from the diagonalization of the covariance matrix. The proportion rapidly decreases and converges to zero with the increasing of PC index in each system. The first two PCs together account for approximately 47.9% and 45.6% of the total variance in the nonmodeled and the modeled systems, respectively. In an equilibrium system, the motions on the backbone are mainly the localized random motions. Thereby, PC1 and PC2 of the two systems capture higher fraction of the system's variance.

**Figure 4:**
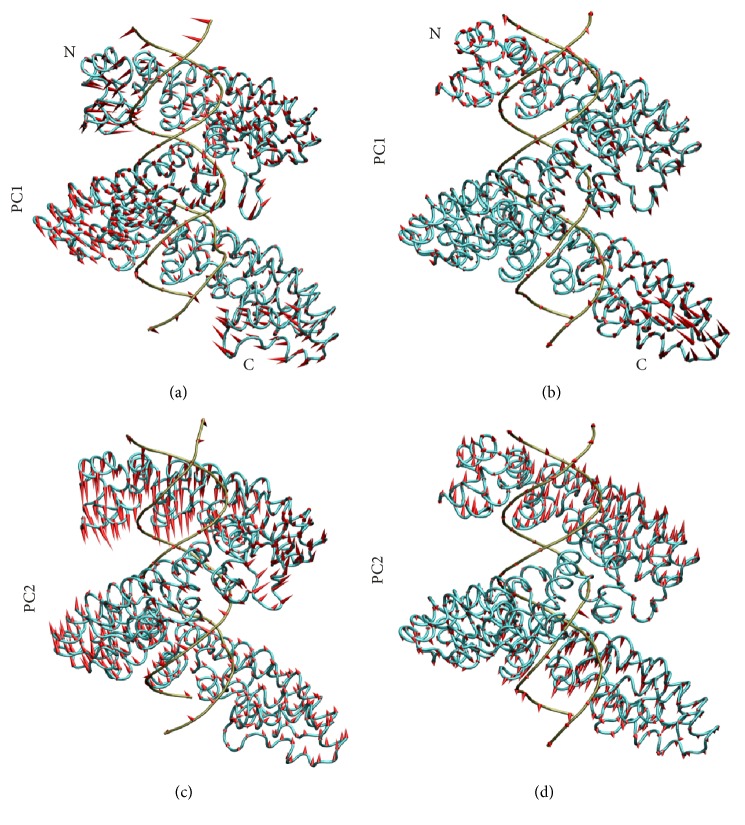
The first and the second slowest motion modes of the nonmodeled system (a and c) and the modeled system (b and d). The average structure is based on the equilibrium trajectories. The length of cone is positively correlated with motive magnitude, and the motive direction is depicted with the orientation of cone.

Figure 4 describes the first and the second slowest motion modes. The first slowest motion exhibits some swing motions towards the DNA major groove in the two systems (Figures 4(a) and 4(b)). By observing their average structures, in the nonmodeled system the last few repeats show a conformation far from the DNA major groove (Figure 4(a)). It is presumably because the swing motion breaks the protein-DNA interaction at the binding interface. In contrast, the protein-DNA interface of the modeled system still keeps a compact conformation at the C-terminus (Figure 4(b)). This conformation difference of the C-terminus between the systems is consistent with the above RMSFs analysis.

**Figure 5:**
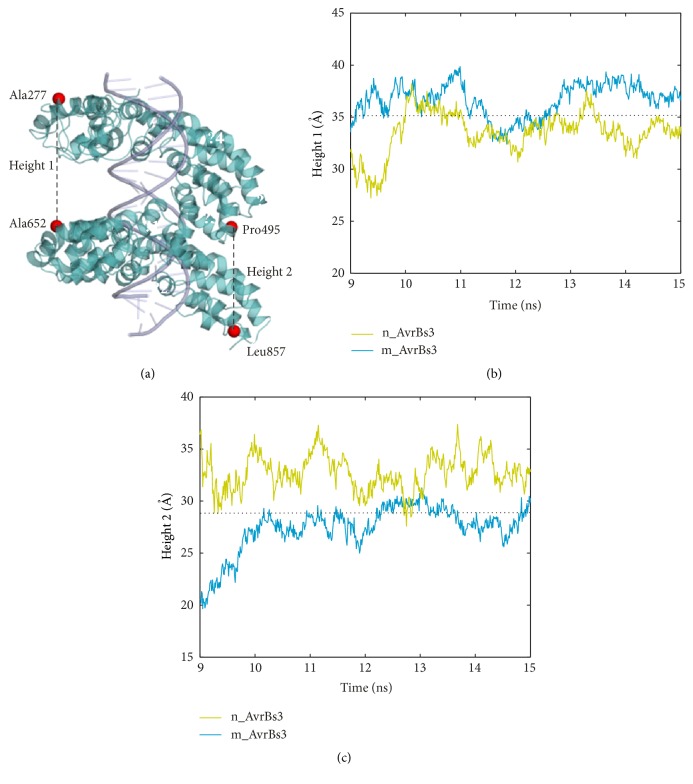
The height change of the superhelical structure of AvrBs3. (a) The height of the first half of the superhelical structure is assessed by the distance between the C*α* atoms of Ala277 and Ala652 and that of the second by the distance between the C*α* atoms of Pro495 and Leu857. (b) The height change of the first half of the superhelical structure versus simulation (solid line) and the value from crystal structure (dotted line). (c) The height change of the second half of the superhelical structure versus simulation (solid line) and the value from crystal structure (dotted line).

The second slowest motion mode shows some extension-compression movements of the superhelical structure of AvrBs3 (Figures 4(c) and 4(d)). The previous X-ray scattering (SAXS) data [7] and crystal structure study [8] revealed that TALEs underwent a compressed conformational change upon DNA interaction. This conformational change caused the height change of the superhelical structure of TALE protein [8]. Then, the four atoms, which are C*α* atoms of Ala277 (repeat 0), Pro495 (repeat 7), Ala652 (repeat 11), and Leu857 (repeat 17), were selected to measure the height change of the first and the second halves of the superhelical structure (Figure 5(a)). For the first half of the superhelical structure, the average height is 35.1 Å, 33.5 Å, and 36.7 Å for the crystal structure, the nonmodeled system, and the modeled system, respectively (Figure 5(b)). For the second half of the superhelical structure, the average height is 28.9 Å, 32.7 Å, and 27.4 Å for the crystal structure, the nonmodeled system, and the modeled system, respectively (Figure 5(c)). As a whole, the modeled system still maintains a compressed conformation relative to the crystal structure. In the nonmodeled system, the superhelical structure of AvrBs3 is comparatively more extended. The combined analyses of the first and the second slowest motions clearly show that the AvrBs3-DNA complex structure keeps a more compact conformation in the presence of the last half repeat. Meanwhile, the increase of structural compactness of TALE is associated with the DNA binding [7, 8]. Therefore, the last half repeat makes an important contribution to the TALE-DNA binding.

### 3.3. Groove Deformation of DNA

**Figure 6:**
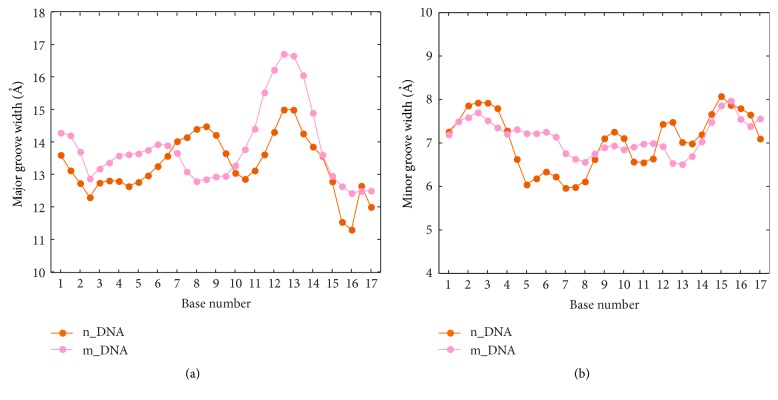
Average values of groove widths calculated from the equilibrium trajectories along the target sequence (from position 1 to position 17) in the nonmodeled (orange) and the modeled (pink) systems. (a) Major groove widths. (b) Minor groove widths.

DNA groove dimensions are important structural feature in processes involving specific protein-DNA binding [32]. Then, the DNA groove parameters of the two systems were calculated by the Curves program [31] from the equilibrium trajectories. The result is shown in Figure 6. Along the target sequence, except for positions 8 and 9, the major groove of the modeled system is almost always wider than that of the nonmodeled system (Figure 6(a)). The wider major groove makes the side chain of the key amino acid of protein more accessible to nucleotide bases and then can mediate more protein-DNA contacts. It is suggested that the efficiency of DNA major groove binding by AvrBs3 should be relatively higher in the modeled system. The interactions at the protein-DNA interface will be analyzed in the next section.

**Figure 7:**
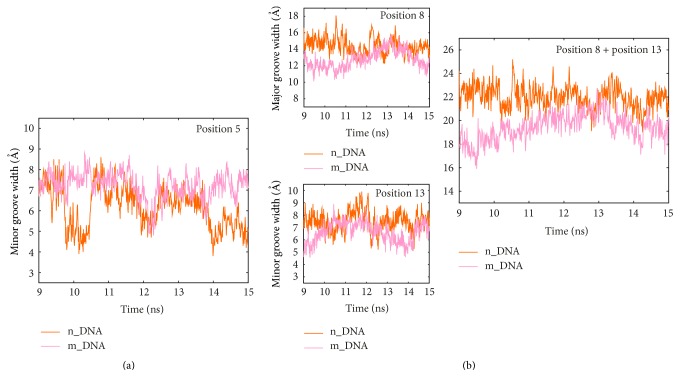
Time-dependent fluctuations of DNA groove widths at positions 5 (a) and 8 and 13 (b) calculated from the equilibrium trajectories in the nonmodeled (orange) and the modeled (pink) systems.

Notably, the major groove at positions 8 and 9 is markedly narrowed in the modeled system relative to the nonmodeled system. To investigate whether there is some relationship between the groove narrowing of DNA and the structural compression of AvrBs3, we compared the time-dependent fluctuation of groove width at each base pair step with the height change of the superhelical structure of AvrBs3. For the first part of the complex structure (Figure 5(a)), the height change of AvrBs3 (Figure 5(b)) is similar to the fluctuation of minor groove width at position 5 (Figure 7(a)). For the second part of the complex structure (Figure 5(a)), the height change of AvrBs3 (Figure 5(c)) accompanies the deformations of major groove at position 8 and of minor groove at position 13 together (Figure 7(b)). It indicates that the TALE-DNA binding process is associated with some structural adaptation of the DNA as well as the AvrBs3 in order to accommodate each other. The conformational difference between the two systems may reflect the changes of the TALE-DNA binding.

### 3.4. Interactions at the Interface

To compare the difference of the protein-DNA interaction between the two systems, we examined the hydrogen bonds along the DNA major groove based on the equilibrium trajectories. The hydrogen bond calculation was performed with VMD 1.9 [21] using a distance cut-off value of 3.5 Å and an angle cut-off value of 45°. The result is listed in Table 1 with occupancy over 30%. Relative to the nonmodeled system, the modeled system has four additional specific hydrogen bonds and four additional nonspecific hydrogen bonds. The calculation of hydrogen bond proves that the modeled system has a higher protein-DNA binding efficiency in the DNA major groove. These additional interactions help the modeled system to achieve higher stability, which is consistent with the above analysis of RMSDs.

**Figure 8:**
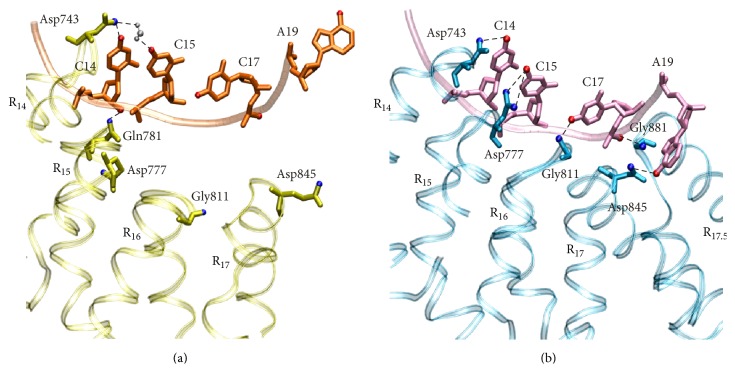
The interactions between the last few repeats and DNA from representative structures in the nonmodeled (a) and the modeled (b) systems. The repeats, DNA, and water molecule are depicted with ribbons, tube, and CPK models, respectively. Repeats 14, 15, 16, 17, and 17.5 are labeled as R_14_, R_15_, R_16_, R_17_, and R_17.5_, respectively. Nucleotide bases cytosine 14, cytosine 15, cytosine 17, and adenine 19 are labeled as C14, C15, C17, and A19, respectively. Thymine 16 and thymine 18 are omitted for clarity.

Compared with the nonmodeled system, the additional specific interactions of the modeled system are mainly formed by the N- and C-terminal repeats, especially by the last few repeats (Table 1).  Figure 8 describes the difference of the specific interaction between the two systems. In the nonmodeled system (Figure 8(a)), Asp743 (repeat 14) forms a direct and a water-mediated hydrogen bond with cytosine 14 and cytosine 15 separately. OE2 of Gln781 (repeat 15) interacts with O3′ of cytosine 14. Meanwhile, repeats 16~17 lose the contact with nucleotide bases. The C-terminal repeats show a conformation far from the backbone of DNA. In the modeled system (Figure 8(b)), Asp743 (repeat 14), Asp777 (repeat 15), Gly811 (repeat 16), and Asp845 (repeat 17) form stable specific hydrogen bonds with cytosine 14, cytosine 15, cytosine 17, and adenine 19, respectively. Notably, N of Gly881 (repeat 17.5) interacts with O1P of cytosine 17. This phosphate binding adopts a compact conformation at the protein-DNA interface and further helps to mediate more base-specific interactions. The previous study revealed that the last repeat is always a truncated half repeat in all natural TALEs [1], but the role of this last half repeat is not clear in the specific binding process of TALE-DNA. Our study indicates that the last half repeat helps to stabilize a compact conformation at the TALE-DNA interface and then indirectly facilitates the specific interactions between TAL repeats and nucleotide bases. Therefore, the last half repeat is required for improving the recognition efficiency of specific DNA sequences by TALE.

## 4. Conclusions

In this study, MD simulations were performed to investigate the role of the last half repeat in the recognition and binding of TALE-DNA. The simulated result indicated that the stability of the modeled system (having the last half repeat) is higher than that of the nonmodeled system (lacking the last half repeat). The PCA analysis revealed that the AvrBs3 structure of the nonmodeled system is more extended in comparison with the crystallographic data. In contrast, the AvrBs3 of the modeled system still keeps the structural compactness. According to the previous experimental studies, this increase of the structural compactness of TALE is associated with the DNA binding. We also compared DNA groove parameters of the two systems. As a whole, the DNA major groove of the modeled system is relatively wider, which allows the side chain of the key amino acid of protein to be more accessible to nucleotide bases. It was suggested that the protein-DNA binding efficiency of the modeled system may be relatively higher. Then, we calculated the hydrogen bonds at the protein-DNA interface. Comparatively, the nonmodeled system loses a considerable number of hydrogen bonds. The modeled system still keeps relatively stable protein-DNA binding. These additional interactions are mainly formed by the N- and C-terminal repeats. In particular, the last half repeat stabilizes the phosphate binding with DNA at the C-terminus and then helps to adopt a compact conformation at the protein-DNA interface. This compact conformation improves the specific recognition efficiency between TAL repeats and nucleotide bases. Our study reveals the important role of the last half repeat in high-efficient recognition of the DNA target sequence by TALE. It provides a deeper understanding of the recognition mechanism of TALE-DNA.

## Figures and Tables

**Table 1:** The hydrogen bonds with occupancy over 30%.

Base	Nonmodeled system			Modeled system		
Position	Protein^(id)^	DNA	HDO^*∗*^	Protein^(id)^	DNA	HDO^*∗*^
0	Gly302-N^(1)^	T0-O2P^*α*^	74.88%	Thr270-N^(0)^	T0-O2P^*α*^	83.19%
				Gln305-N^(1)^	T0-O1P^*α*^	63.73%
1	***Asp301_OD2*** ^**(*1)***^	***A1-N6*** ^**α**^	***39.41%***	***Asp301_OD1*** ^**(*1)***^	***A1-N6*** ^**α**^	***60.35%***
				***Asp301_OD1*** ^**(*1)***^	***A1-N7*** ^**α**^	***58.47%***
	Gln339-NE2^(2)^	A1-O1P^*α*^	50.89%	Gln339-NE2^(2)^	A1-O1P^*α*^	88.85%
2				***Asp301_OD1*** ^**(*1)***^	***T2-O4*** ^**α**^	***47.65%***
	Gln373-NE2^(3)^	T2-O1P^*α*^	35.44%	Gln373-NE2^(3)^	T2-O1P^*α*^	67.05%
3	Gln407-NE2^(4)^	A3-O2P^*α*^	53.24%	Gln407-NE2^(4)^	A3-O2P^*α*^	58.90%
4	Gln441-NE2^(5)^	T4-O1P^*α*^	63.73%	Gln441-NE2^(5)^	T4-O1P^*α*^	86.36%
5	Gln475-NE2^(6)^	A5-O2P^*α*^	30.78%	Gln475-NE2^(6)^	A5-O2P^*α*^	45.92%
6	Gln509-NE2^(7)^	A6-O2P^*α*^	63.56%	Gln509-NE2^(7)^	A6-O2P^*α*^	79.03%
7	Gln543-NE2^(8)^	A7-O2P^*α*^	96.01%	Gln543-NE2^(8)^	A7-O2P^*α*^	94.18%
8	**Asp539-OD2** ^(8)^	**C8-N4** ^**α**^	**30.23%**	**Asp539-OD2** ^(8)^	**C8-N4** ^**α**^	**35.44%**
	Gln577-NE2^(9)^	C8-O1P^*α*^	92.68%	Gln577-NE2^(9)^	C8-O1P^*α*^	63.73%
9	**Asp573-OD1** ^(9)^	**C9-N4** ^**α**^	**36.77%**	**Asp573-OD1** ^(9)^	**C9-N4** ^**α**^	**34.11%**
	**Asp573-OD2** ^(9)^	**C9-N4** ^**α**^	**30.28%**	**Asp573-OD2** ^(9)^	**C9-N4** ^**α**^	**60.90%**
	Gln611-NE2^(10)^	C9-O1P^*α*^	54.08%	Gln611-NE2^(10)^	C9-O1P^*α*^	90.18%
10	Gln645-NE2^(11)^	T10-O1P^*α*^	88.19%	Gln645-NE2^(11)^	T10-O1P^*α*^	88.85%
11	Gln679-NE2^(12)^	A11-O2P^*α*^	73.21%	Gln679-NE2^(12)^	A11-O2P^*α*^	88.52%
12	Gln713-NE2^(13)^	A12-O2P^*α*^	88.69%	Gln713-NE2^(13)^	A12-O2P^*α*^	81.70%
13				Gln747-NE2^(14)^	C13-O1P^*α*^	57.90%
14	**Asp743-OD2** ^(14)^	**C14-N4** ^**α**^	**64.73%**	**Asp743-OD2** ^(14)^	**C14-N4** ^**α**^	**85.69%**
	**Gln781-OE2** ^(15)^	**C14-O3** ^′**α**^	**32.78%**			
15	***Asp743-OD2*** ^(14)^	***C15-N4*** ^**α**^	***60.12%***	**Asp777-OD1** ^(15)^	**C15-N4** ^**α**^	**61.40%**
				**Asp777-OD2** ^(15)^	**C15-N4** ^**α**^	**37.27%**
	Lys814-NZ^(16)^	C15-O1P^*α*^	57.90%	Lys814-NZ^(16)^	C15-O2P^*α*^	99.83%
16				Lys848-NZ^(17)^	T16-O1P^*α*^	83.86%
17				**Gly811-O** ^(16)^	**C17-N4** ^**α**^	**88.02%**
				Gly881-N^(17.5)^	C17-O1P^*α*^	35.62%
19				**Asp845-OD1** ^(17)^	**A19-N6** ^**α**^	**43.43%**

^id^The index of a repeat that a residue belongs to.

^*∗*^HDO is the abbreviation of hydrogen bond occupancy.

^*α*^DNA base belonging to the sense strand of DNA.

Hydrogen bonds in bold and nonbold reflect the specific and nonspecific interactions, respectively. Bold in italics denotes the specific and water-mediated hydrogen bonds.
